# Ideological differences in the expanse of the moral circle

**DOI:** 10.1038/s41467-019-12227-0

**Published:** 2019-09-26

**Authors:** Adam Waytz, Ravi Iyer, Liane Young, Jonathan Haidt, Jesse Graham

**Affiliations:** 1 Northwestern University, 2211 Campus Dr, Evanston, IL 60208 USA; 2 Facebook, 12721W Jefferson Blvd, Los Angeles, CA 90066 USA; 3 Boston College, Gasson Hall, 140 Commonwealth Avenue, Chestnut Hill, MA 02467 USA; 4 New York University, Kaufman Management Center, 44 West Fourth Street, 7-98, New York, NY 10012 USA; 5 University of Utah, Spencer Fox Eccles Business Building, 1655 East Campus Center Drive, Salt Lake City, UT 84112 USA

**Keywords:** Politics, Human behaviour

## Abstract

Do clashes between ideologies reflect policy differences or something more fundamental? The present research suggests they reflect core psychological differences such that liberals express compassion toward less structured and more encompassing entities (i.e., universalism), whereas conservatives express compassion toward more well-defined and less encompassing entities (i.e., parochialism). Here we report seven studies illustrating universalist versus parochial differences in compassion. Studies 1a-1c show that liberals, relative to conservatives, express greater moral concern toward friends relative to family, and the world relative to the nation. Studies 2a-2b demonstrate these universalist versus parochial preferences extend toward simple shapes depicted as proxies for loose versus tight social circles. Using stimuli devoid of political relevance demonstrates that the universalist-parochialist distinction does not simply reflect differing policy preferences. Studies 3a-3b indicate these universalist versus parochial tendencies extend to humans versus nonhumans more generally, demonstrating the breadth of these psychological differences.

## Abstract (web summary)

How do liberals and conservatives differ in their expression of compassion and moral concern? The authors show that conservatives tend to express concern toward smaller, more well-defined, and less permeable social circles, while liberals express concern toward larger, less well-defined, and more permeable social circles.

## Introduction

In 2006, then Democratic Senator Barack Obama bemoaned the country’s “empathy deficit,” telling college graduates, “I hope you choose to broaden, and not contract, your ambit of concern.” In 2012, Republican presidential challenger Mitt Romney said, “President Obama promised to begin to slow the rise of the oceans and heal the planet. My promise is to help you and your family.”

The distinction between Obama and Romney captures the distinct worldviews of American political liberals and conservatives, respectively. Romney prioritized the family unit, whereas Obama highlighted the planet broadly. This difference in parochialism versus universalism became exacerbated during the 2016 presidential election, with one article noting, “Trump vs. Hillary Is Nationalism vs. Globalism, 2016^1^,” contrasting the more parochial Republican candidate with the more universalist Democratic candidate. Others have characterized the Trump administration’s policy decisions as battles between nationalists (typified by parochialism) and globalists (typified by universalism)^2^.

These differential tendencies toward parochialism and universalism on the political right and left, respectively, extend beyond the United States as well. For example, leading French right-wing politician, Marie Le Pen declared in 2016, “The gap is not between the Left and the Right, but between globalists and patriots. The globalists are acting for the dilution of France and its people in a huge worldwide magma. The patriots hope that the nation constitutes the most protective space for the French^3^.” Across Western Europe, ideological battles between the left and right have centered on this tension between universalism and parochialism.

*Universalism* refers to moral regard directed toward more socially distant and structurally looser targets, relative to socially closer and structurally tighter targets. *Parochialism* refers to moral regard directed toward socially closer and structurally tighter targets, relative to socially more distant and structurally looser targets. Universalist moral circles and parochial moral circles in this context are concentric, with one encompassing the other. These circles refer to groups of targets toward which one expends moral regard, and reflect the concept of moral circles popularized by Singer^4^ (see also Burke^5^). They are akin to the idea of moral communities that comprise one’s in groups (discussed by Deutsch et al.^6,7^), in which entities can be included or excluded as worthy of moral regard, as well as to concentric circles of identity defined by self-categorization theory (whereby one’s self-concept can include increasingly distant social groups depending on one’s level of abstraction)^8^. While “parochial” sometimes has a negative connotation, we do not imply any such evaluation here and simply use it to describe maintaining a tight (versus loose) moral circle.

Previous research supports this universalist–parochial distinction between liberals and conservatives^9^. For instance, conservatives, relative to liberals, express greater need for closure, order, and structure^10–12^. Personality research shows social liberals consistently score higher on openness, whereas social conservatives score higher on conscientiousness^13^. Taken together, existing work suggests that political conservatism reflects a greater tendency to seek structure, to avoid ambiguity, changes to the status quo, and novelty. By this account, political liberalism represents greater comfort with lack of structure, new experiences, and novel information.

Given ideological differences in open versus closed styles of information processing, moral concern might follow a similar pattern. In prioritizing closure, order, and stability, conservatives should express concern toward smaller, more well-defined, and less permeable social circles (relative to broader ones). In prioritizing openness, tolerance for ambiguity, and desire for change, liberals should express concern toward larger, less well-defined, and more permeable social circles (relative to smaller ones).

Beyond low-level cognitive and motivational differences, one additional line of work supports the ideological distinction between parochial–universalist differences in compassion. This line of research stems from Moral Foundations Theory (MFT)^14–16^, which characterizes liberals and conservatives as diverging along two classes of intuitive moral values. Liberals care about harm and fairness (individualizing values), whereas conservatives care more about loyalty, authority, and sanctity (binding values). This research again suggests a differing focus such that liberals tend to express compassion toward individuals broadly construed, whereas conservatives emphasize compassion toward their immediate social groups. Supporting this idea, separate work indeed found that endorsement of individualizing values is positively correlated with moral expansiveness (moral consideration for entities, including plants and animals, beyond one’s immediate in group) whereas endorsement of binding values is negatively correlated with moral expansiveness^17^.

The present research provides empirical evidence for these differing ideological patterns of compassion and extends these patterns to stimuli across a range of measures. This work also shows these broader ideological differences are rooted in perceptual differences. These differences appear to stem also from a broader historical trend that has accelerated in recent decades as most countries have become wealthier and safer. Christian Welzel, a lead researcher for the World Values Survey, has described how reduced “existential threats” change values:

Fading existential pressures open people’s minds, making them prioritize freedom over security, autonomy over authority, diversity over uniformity, and creativity over discipline… the existentially relieved state of mind is the source of tolerance and solidarity beyond one’s in group^18^.

Our research is consistent with Welzel’s characterization of the general shift from “survival values” that increase dependence on close others, to “emancipative values” that downplay local ties—and loyalties—and lead people to look farther afield for social relationships.

These studies aim to connect Singer’s^4^ idea of the moral circle to empirical political psychology. Beyond demonstrating a universalist–parochial distinction between liberals and conservatives, this research examines whether this distinction reflects mere political preferences, or something deeper. Universalism may reflect favorability toward policies that promote open borders (and encourage immigration) and that promote diplomacy toward ostensibly hostile nations. Such policies represent extending moral regard beyond one’s immediate group (e.g., the nation) and to the world more broadly. Similarly, parochialism may reflect favorability toward stricter immigration policies and defense spending to protect one’s nation—these policies represent prioritizing the well-being of one’s own nation at the potential expense of others. On the other hand, if the universalist–parochial distinction reflects a worldview beyond policy interests, then it should reflect evaluations of stimuli completely devoid of social or political relevance, for example abstract, animate shapes. Thus, we tested whether liberals and conservatives would display universalist and parochialist tendencies, respectively, in terms basic perceptual preferences. Finally, we examined whether this universalist–parochialist difference would map on to moral concern for humans exclusively versus a broader conception of the moral universe that includes nonhumans as well. Importantly, this work uses both measures developed for this work that explicitly capture the expanse of one’s moral circle as well as established measures that assess moral consideration for specific targets, to provide convergent evidence across studies.

Studies 1a–1c examine universalist versus parochial differences in the domains of friends versus family (friends typically constitute a larger, broader and more diffuse group than family) and the world versus the nation (the world encompasses one’s nation). Studies 2a–2b show this universalist–parochial distinction maps on to abstract entities (animated shapes) distinguished only by low-level perceptual properties. Studies 3a–3b demonstrate that this universalist–parochial distinction maps on to moral concern for humans exclusively compared with a social world that includes nonhumans. Across studies, we predicted that liberalism versus conservatism would be associated with universalism relative to parochialism, even in the context of preference for shapes devoid of social relevance and humans versus nonhumans.

## Results

For all results in this paper, *p*-values that follow the label *r* were generated from Pearson’s correlations and *p*-values that follow standardized betas were generated from *t* -tests on regressions. All other statistical tests are specified.

**Fig. 1:**
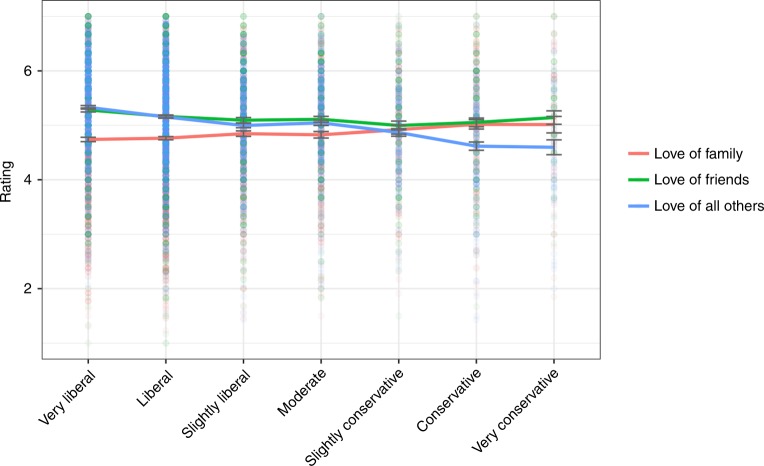
Love by political ideology, Study 1a. Error bars represent standard errors, solid lines indicate means. Source data are provided as a Source Data file

Study 1a tests the hypothesis that liberalism and conservatism would correlate with love for friends and love for family, respectively. The results showed conservatism was positively related to love of family, *r* (3,362) = 0.065, *p* < 0.001, and negatively related to love of friends, *r* (3,360) = −0.065, *p* < 0.001 (for means, see Fig. 1) (see Supplementary Note 2 for exploration of quadratic effects). Admittedly, these correlations are exceedingly small and should be interpreted with caution. Critically, however, Steiger *z* -tests conducted on participants who had scores for each of these scales demonstrated that these correlations differed from each other (zs > 2.87, *ps* < 0.004). In addition, conservatism was unrelated to romantic love (*r* = −0.01, *p* = 0.68) (a construct that combines friendship and family relations) and was negatively correlated with love for all others, *r* (3,362) = −0.20, *p* < 0.001—this result suggests liberalism is associated with a more universalist sense of compassion.

Separate multiple regressions in which romantic love, love of friends, love of family, and love of all others were the outcome variables and political ideology, age, gender, and education were predictor variables revealed that the effect of political ideology remained the same—significant for love of friends, love of family, love of all others, and nonsignificant for romantic love (see Table 1 for standardized betas). These analyses suggest that political ideology meaningfully affects love of friends, family, and others universally, independent of other related demographic variables.

**Table 1:** Standardized betas for regressions using political ideology, education, age, and gender

Study	Outcome measure	Political ideology	Education	Age	Gender
1a	Romantic love	0.01	0.05*	−0.02	−0.10**
1a	Love of family	0.10**	0.05**	−0.07**	−0.14**
1a	Love of friends	−0.05**	−0.01	−0.015	−0.17**
1a	Love of all others	−0.17**	0.01	0.175**	−0.19**
1b	Nationalism	0.45**	−0.06**	0.19**	−0.035**
1b	Universalism	−0.42**	−0.06**	0.18**	−0.13**
1c	Identification with community	0.08**	0.06**	0.085**	−0.13**
1c	Identification with country	0.285**	0.01	0.15**	−0.10**
1c	Identification with all humanity	−0.33**	−0.03**	0.04**	−0.155**
2a	Preference for looseness versus tightness	−0.20**	0.01	−0.02	0.003
2a	Preference for color diversity	−0.03^+^	−0.04*	0.23**	−0.05**
2b	Preference for looseness versus tightness	−0.12**	0.02	−0.06*	0.03
2b	Preference for circle versus triangle	−0.07**	−0.04	−0.01	−0.05^+^
3a	Personal moral allocation to humans	0.34**	−0.01	−0.09	0.12
3a	Ideal moral allocation to humans	0.28**	−0.01	−0.04	0.18*
3a	Weighted personal moral circle	−0.35**	−0.03	0.07	−0.11
3a	Weighted ideal moral circle	−0.26**	−0.02	0.01	−0.15^+^
3b	Proportion moral allocation to humans	0.13*	0.15*	0.08	0.13*
3b	Total moral allocation	0.055	0.03	−0.01	−0.09
3b	Moral allocation to humans	0.056	0.03	−0.01	−0.09
3b	Moral allocation to nonhumans	0.055	0.03	−0.01	−0.09

Notes: ^+^*p* < 0.09; *p < 0.05; **p < 0.01. Effect of gender for moral allocation to humans (Study 3b) becomes marginally significant (*p* = 0.062) when including the one participant whose total allocation falls outside of 3SD of the mean

**Fig. 2:**
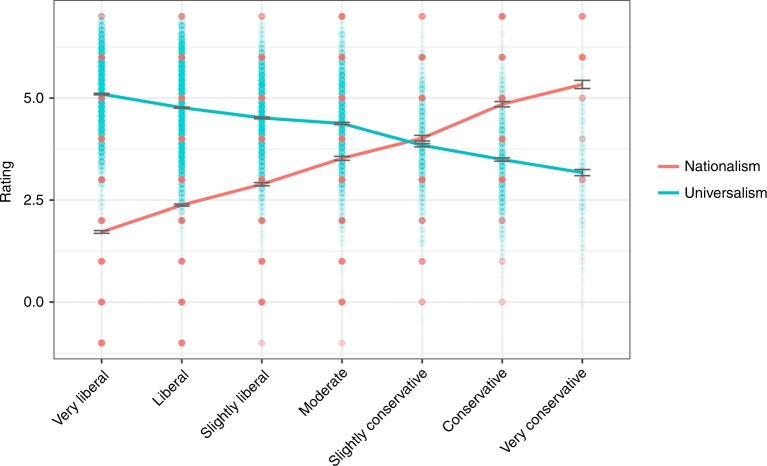
Endorsement of values by political ideology, Study 1b. Error bars represent standard errors, solid lines indicate means. Source data are provided as a Source Data file

Study 1b tests the hypothesis that just as liberalism and conservatism will correspond to valuing the world and valuing the nation, respectively. Conservative ideology was negatively correlated with universalism, *r* (13,154) = −0.41, *p* < 0.001, again demonstrating that conservatism is negatively related to a universal love of others, whereas liberalism is positively related to this sense of universal compassion. In addition, conservative ideology was positively correlated with nationalism, *r* (13,030) = 0.46, *p* < 0.001 (see Fig. 2 for means). A Steiger *z* test on participants who had scores on both of these measures demonstrated that these correlations differed significantly from one another (*z* = 77.04, *p* < 0.001).

Separate multiple regressions in which universalism and nationalism were the outcome variables, respectively and political ideology, age, gender, and education were predictor variables, revealed that the effect of political ideology persisted (see Table 1). Liberalism continued to predict universalism significantly whereas conservatism continued to predict nationalism significantly. These findings suggest that political ideology meaningfully affects universalism and nationalism, independent of other related demographic variables.

**Fig. 3:**
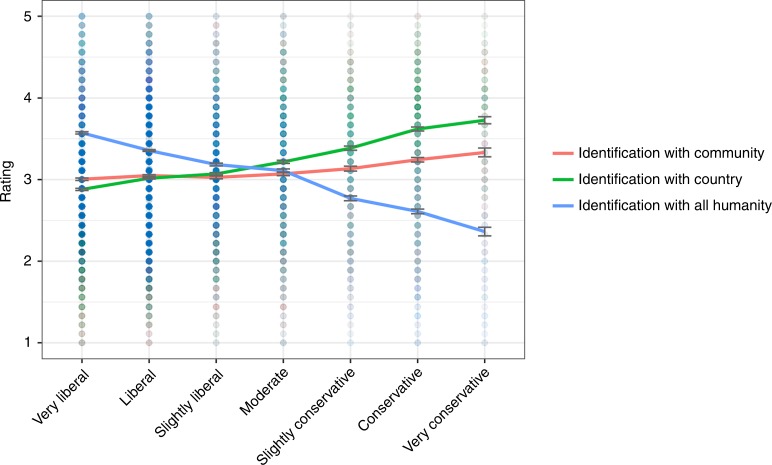
Identification by political ideology, Study 1c. Error bars represent standard errors, solid lines indicate means. Source data are provided as a Source Data file

Like Study 1b, Study 1c tests the hypothesis that conservatism corresponds to a more parochial or national sense of compassion whereas liberalism corresponds to a universal sense of compassion. Conservatism correlated with identification with country, *r* (14,176) = 0.28, *p* < 0.001 a liberalism correlated with identification with the world, *r* (14,176) = −0.34, *p* < 0.001. In addition, conservatism showed a small but significant correlation with identification with community, *r* (14,176) = 0.074, *p* < 0.001. Steiger *z* tests demonstrated that the correlations differed significantly for community and country (*z* = 26.74, *p* < 0.001), for country and all humans (*z* = 67.09, *p* < 0.001), and for community and all humans (*z* = 43.95, *p* < 0.001) (for means, see Fig. 3).

Separate multiple regressions in which identification with community, identification with country, and identification with all humanity were the outcome variables and political ideology, age, gender, and education were predictor variables revealed that the effect of political ideology remained the same (see Table 1). These analyses suggest that political ideology meaningfully affects identification with community, country, and all humans, independent of other related demographic variables.

Studies 1a–1c demonstrate that conservatives are more parochial than liberals—their moral circles are more constrained. This political difference manifests at the level of family versus friends and the nation versus the world. These differences are perhaps unsurprising given well-known policy disagreements on issues affecting these specific circles of family, friends/community, nation, and world^16,19,20^. If ideological differences in compassion simply reflect policy issue differences, then they should affect attitudes toward targets relevant to these social issues. However, if these ideological differences permeate more deeply into liberals and conservatives general worldviews they should manifest in evaluations of targets completely devoid of social and political relevance. We test this possibility in Studies 2a and 2b.

Study 2a tested whether conservatives (relative to liberals) would prefer tight (relative to loose) geometric structures. We further predicted that these differing preferences would correspond to compassion toward social circles (that involved specifically human targets) examined in Studies 1a–1c. Conservatism was associated significantly with preference for tightness relative to looseness in geometric structures, *r* (4426) = −0.20, *p* < 0.001. These results suggest, as predicted, conservatism relative to liberalism corresponds to a preference for tighter structures even when devoid of social relevance.

We also predicted, a priori, that liberals would show a preference for color diversity (i.e., the different colors represented by the geometric structures) across stimuli, but found no significant correlation, *r* = −0.01, *p* = 0.35. This finding suggests that ideology specifically relates to preference for the movement patterns of the structures, and not more broadly related to their homogeneity or heterogeneity.

Separate multiple regressions in which preference for looseness-tightness and preference for diversity of color were the outcome variables, respectively, and political ideology, age, gender, and education were predictor variables revealed that political ideology continued to predict looseness-tightness preference significantly. In addition, a multiple regression revealed that preference for diversity was in the predicted direction (associated positively with liberal ideology), but the effect was tiny and only of marginal significance (*p* = 0.078) (see Table 1). These analyses suggest that political ideology meaningfully affects this basic preference for geometric looseness-tightness, independent of other related demographic variables.

Ideology could be linked to geometric preferences in ways completely unrelated to the link between ideology and social preferences. We therefore examined whether this basic preference for geometric structure maps on to social judgments, testing whether this preference helps account for the relationship between political ideology and moral regard for tight versus loose social structures examined in previous studies.

We tested this by capitalizing on a unique subset of participants who—in addition to completing this study—had also completed one of Studies 1a, 1b, and 1c. These participants enabled us to examine the association between scores on the present geometric shapes task and a social looseness-tightness score reflecting participants’ preference for small social circles (i.e., family and the nation) relative to larger social circles (i.e., friends and the world, respectively).

For each participant, we computed a social looseness–tightness score by first standardizing all measures in Studies 1a–1c, and then averaging scores for explicitly “tight” circles and subtracting this average from the average of scores for explicitly “loose” circles. In other words, we computed the average of standardized scores for the love of family scale (Study 1a), the national security subscale (Study 1b), and the identification with country subscale (Study 1c) (“tight measures”), and computed the average of standardized scores for the love of friends and love for all other subscales (Study 1a), the universalism subscale (Study 1b), and the identification of all humanity subscale (Study 1c) (“loose measures”). We then subtracted the average of loose measures from the average of tight measures as follows:

(average (love for friends_Study1a_, love for all others_Study1a_, value of universalism_Study1b_, identification with all humanity_Study1c_)) − (average (love of family_Study1a_, value of national security_Study1b_, identification with country_Study1c_)).

To maximize statistical power, we included people who did not have scores for all measures in the equation, although scores were not computed for people who only had scores for the tight or loose side of the equation, leaving 921 participants. In other words, this score reflected participants’ moral regard for friends and global humanity relative to family and one’s nation.

We then used bootstrapping mediation analysis using the SPSS PROCESS macro^21^ (bias-corrected, 20,000 resamples) to examine whether preference for geometric looseness-tightness mediates the relationship between political ideology and social looseness-tightness. This analysis confirmed partial mediation, in that political ideology indirectly affected people’s preference for social looseness–tightness through a preference for geometric looseness–tightness (95% confidence interval = −0.02 to −0.0002).

Thus, at very least, the relationship between ideology and preference for geometric looseness–tightness is related to preference for social looseness–tightness, and this more “primitive” preference for looseness–tightness might drive people of different political ideologies toward social circles of different expansiveness. Most important, this study demonstrates that the looseness-tightness preference is not limited to circles with which people have preexisting associations, and this perceptual preference is linked to a preference for more well-defined tight versus loose social circles. Study 2b provides a conceptual replication to examine these effects further.

Study 2b is a conceptual replication of Study 2a that again manipulated tightness versus looseness. Conservatism was associated significantly with preference for tightness relative to looseness, *r* (2072) = −0.15, *p* < 0.001. These results suggest that again, as predicted, conservatism relative to liberalism corresponds to an overall preference for tighter structures even when these structures are devoid of social relevance.

As we presented geometric structures of different shapes, we had also predicted that conservatives would prefer the shape of a triangle more often than liberals, and liberals would prefer the circle more often than conservatives (because it is the most “egalitarian” shape, with no dot seeming more important than any other). This prediction was confirmed, marginally: conservatives relative to liberals slightly preferred the triangle relative to the circle, *r* (2072) = −0.04, *p* = 0.054.

Separate multiple regressions in which preference for looseness-tightness and preference for circle were the outcome variables, respectively, and political ideology, age, gender, and education were predictor variables revealed that political ideology predicted looseness-tightness preference and preference for shape significantly (see Table 1). These analyses again suggest that political ideology meaningfully affects this basic preference for geometric looseness-tightness, independent of other related demographic variables.

Again, to examine the relationship between ideology, geometric looseness–tightness preference, and social looseness–tightness preference, we computed the same social looseness–tightness score as in Study 2a and conducted the same mediation analysis as in Study 2a. This analysis, with 679 participants, also confirmed partial mediation—political ideology indirectly affected people’s preference for social looseness–tightness through a preference for geometric looseness–tightness (95% confidence interval = −0.04 to −0.095). These findings again suggest that the relationship between ideology and geometric looseness–tightness maps on to preferences for social looseness–tightness.

Building on Studies 1–2, showing that liberals and conservatives demonstrate universalism versus parochialism, respectively, Study 3 tests whether this pattern extends to evaluations of nonhumans versus humans, testing the hypothesis that liberals relative to conservatives will show more moral concern toward nonhuman (relative to human) targets. Although existing work shows ideologies associated with conservatism like social dominance orientation and right-wing authoritarianism predict beliefs in human superiority over nonhuman animals and positive attitudes toward animal exploitation^22,23^, the studies here explicitly test the relationship between political ideology and moral concern toward humans versus nonhumans.

We analyzed separately participants’ ideal and personal allocations of moral regard (measured through “points” described to participants as “moral units”) to different social circles, some of which were clearly human (e.g., family) and some of which were nonhuman (e.g., plants and animals). Political conservatism correlated with actual moral allocation to humans only, *r* (129) = 0.32, *p* < 0.001, and ideal moral allocation to humans only, *r* (129) = 0.26, *p* = 0.003. Allocation to humans only is directly inversely correlated with allocation to nonhumans, so correlations of the same magnitude emerged in the opposite direction for allocation to nonhumans.

**Fig. 4:**
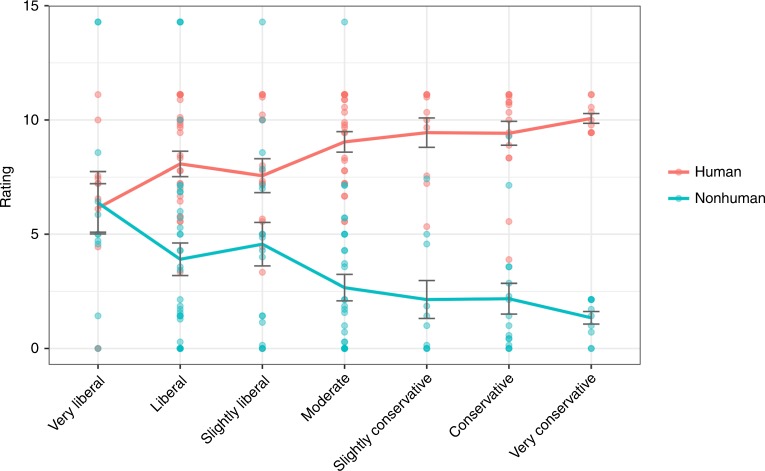
Personal moral allocation to humans and nonhumans by political ideology, Study 3a. Error bars represent standard errors, solid lines indicate means. Source data are provided as a Source Data file

As Fig. 4 shows, the more liberal people were, the more they allocated equally to humans and nonhumans. The further to the right on the ideological spectrum people were, the more likely they were to morally prioritize humans over nonhumans.

We also computed a weighted circle score for each participant by multiplying the numerical rank of each category by the allocation to that category and summing these values. That is, we multiplied “immediate family” by 1, “extended family” by 2…“all things in existence” by 16, and summed the values—larger scores indicated larger moral circles. The significant correlation between ideology and this weighted circle score (*r* (129) = −0.33, *p* < 0.001; *r* (129) = −0.24, *p* = 0.005 for ideal allocation), again demonstrates that as conservatism increases, the extent of the moral circle decreases.

Separate multiple regressions using personal moral allocation to humans, ideal moral allocation to humans, weighted personal circle score, and weighted ideal circle score as outcome variables, with political ideology, age, gender, and education as predictor variables, revealed the same significant effects for political ideology in all cases (see Table 1). These analyses suggest that political ideology meaningfully affects moral allocation independent of related demographic variables.

**Fig. 5:**
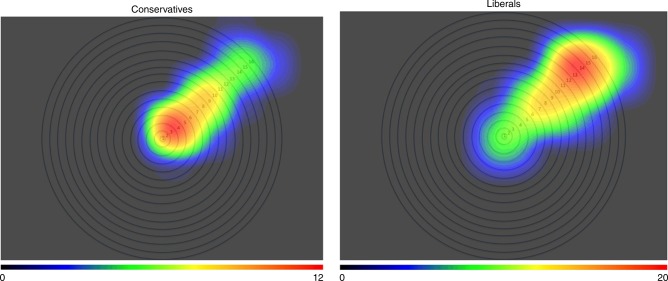
Heatmaps indicating highest moral allocation by ideology, Study 3a. Source data are provided as a Source Data file. *Note*. The highest value on the heatmap scale is 20 units for liberals, and 12 units for conservatives. Moral circle rings, from inner to outer, are described as follows: (1) all of your immediate family, (2) all of your extended family, (3) all of your closest friends, (4) all of your friends (including distant ones), (5) all of your acquaintances, (6) all people you have ever met, (7) all people in your country, (8) all people on your continent, (9) all people on all continents, (10) all mammals, (11) all amphibians, reptiles, mammals, fish, and birds, (12) all animals on earth including paramecia and amoebae, (13) all animals in the universe, including alien lifeforms, (14) all living things in the universe including plants and trees, (15) all natural things in the universe including inert entities such as rocks, (16) all things in existence

Finally, we assessed the heatmaps generated by participants’ clicks on the rung they felt best represented the extent of their moral circle. These qualitative results also demonstrated that liberals (individuals who selected 1, 2, or 3 on the ideology measure) selected more outer rungs, whereas conservatives (individuals who selected 5, 6, or 7 on the ideology measure) selected more inner rungs (see Fig. 5). Overall, these results suggest conservatives’ moral circles are more likely to encompass human beings, but not other animals or lifeforms whereas liberals’ moral circles are more likely to include nonhumans (even aliens and rocks) as well. Study 3a revealed these patterns also when asking about participants’ ideal moral circles. This suggests that both liberals and conservatives, although differing in their moral allocations, feel that their pattern of allocation is the ideal way to adjudicate moral concern in the world.

One caveat to Study 3a is that we constrained the number of units that participants could assign to each group, forcing participants to distribute moral concern in a zero-sum fashion (i.e., the more concern they allocate to one circle, the less they can allocate to another circle). Although research suggests that people indeed do distribute empathy and moral concern in a zero-sum fashion^24–26^, this feature of Study 3a imposes an artificial constraint. Therefore, to examine whether a similar pattern would emerge without this constraint, we conducted Study 3b to test whether the effect would replicate using unlimited units.

**Fig. 6:**
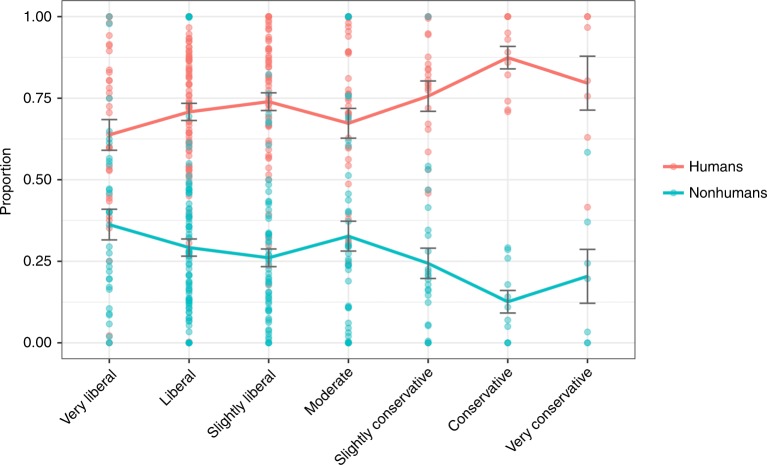
Proportion of moral allocation by ideology, Study 3b. Error bars represent standard errors, solid lines indicate means. Source data are provided as a Source Data file

Study 3b is a conceptual replication of Study 3a, allowing participants unlimited moral units to distribute to various circles. Conservatism was positively correlated with the human allocation proportion score only, *r* (261) = 0.14, *p* = 0.025, and hence negatively with the nonhuman allocation proportion score (for means, see Fig. 6). A multiple regression using the human allocation proportion score as an outcome variable with political ideology, age, gender, and education as predictor variables revealed the same significant effect for political ideology (see Table 1). Thus, even when participants’ allocations were not constrained, the same pattern replicated such that liberals distribute empathy toward broader circles and conservatives distribute empathy toward smaller circles.

Importantly, in addition to examining proportion, we also examined total allocation, and allocation to humans and to nonhumans. Liberals and conservatives did not differ such that political ideology was not significantly correlated with total allocation to all targets, *r* (261) = 0.04, *p* = 0.51, total allocation to humans, *r* (261) = 0.04, *p* = 0.50, or total allocation to nonhumans, *r* (261) = 0.04, *p* = 0.51 (this pattern of the results was the same when excluding the one participant whose allocations fell outside of 3SD of the mean; see below). Separate multiple regressions using these total allocation scores as outcome variables with political ideology, age, gender, and education as predictor variables revealed the same nonsignificant effects for political ideology (see Table 1). Again, these findings demonstrate that liberals and conservatives differ not in the total amount of moral regard per se but rather they differ in their patterns of how they distribute their moral regard.

## Discussion

Seven studies demonstrated that liberals relative to conservatives exhibit universalism relative to parochialism. This difference manifested in conservatives exhibiting greater concern and preference for family relative to friends, the nation relative to the world, tight relative to loose perceptual structures devoid of social content, and humans relative to nonhumans.

Others have identified this universalist–parochial distinction, with Haidt^27^, for example, noting “Liberals…are more universalistic…Conservatives, in contrast, are more parochial—concerned about their groups, rather than all of humanity.” The present findings comprehensively support this distinction empirically, explicitly demonstrating the relationship between ideology and universalism versus parochialism, assessing judgments of multiple social circles, and providing converging evidence across diverse measures.

The present research also leaves several open questions for future examination. Given that we elicited responses from participants rather than examining their spontaneous tendencies toward parochialism or universalism, additional research can assess whether these patterns of moral concern appear even when unprompted. Research examining language use on Twitter supports the parochialism-universalism distinction established here, demonstrating Republicans discuss topics more related to the nation, whereas Democrats discuss topics more pertinent to outside of the home nation^28^. An additional limitation of the present research is that we imposed the distinction of universalist versus parochial on particular circles, and participants might construe these categorizations differently. For example, liberals might construe their friends as more of an inner circle than their family, even though in our operationalization, we treat friends as a more universalist circle and family as a more parochial circle^17^. We imposed distinctions based on objective standards of kinship (family vs. friends) and nationality (nation vs. world), but different patterns of the results might emerge using different parochial–universalist comparisons between different circles (e.g., nation vs. friends).

A separate issue concerns causality. Although our research follows the predominant norms in political ideology research in treating ideology at the independent variable, in some cases ideology could also serve as the dependent variable. That is, moral regard toward particular social circles could alter one’s political ideology over time as well. Rather than a dichotomous chicken-or-egg question (does ideology cause differences in concern, or do differences in concern cause ideology?), the relations between ideology and ambit of concern are likely a complex interplay across development involving interactions between genetic and environmental factors^29^. Both one’s ambit of concern and political ideology are likely preceded by basic temperamental differences demonstrated by research in developmental psychology^30^ and political science^31^. Future work can delineate these causal pathways.

An additional question regarding causality concerns the relationship between low-level perceptual differences in looseness-tightness that emerge in Studies 2a–2b and differences in broader social tendencies toward parochialism-universalism. Although again we cannot fully establish causality, we believe these perceptual preferences for tightness versus looseness reflect basic differences in information processing (desire for closure and structure versus desire for openness), which in turn drive preferences for tighter versus looser social circles. In this way, our work is similar to work showing that left–right ideological differences manifest in basic information processing differences in orientation toward appetitive versus aversive stimuli^32^ and exploration versus non-exploration toward novel stimuli^33^ (even when these stimuli are devoid of social meaning). Our work also demonstrates specific perceptual differences (in Studies 2a–2b) and connects these more fundamental information processing differences to social preferences, moral foundations, and moral expansiveness broadly.

An additional direction for future research is to examine the relationship between ambit of concern and ideology beyond a simple liberal–conservative distinction. Although we employ a general measure of ideology and demonstrate that economic and social ideology reveal the same patterns of the results, in some circumstances social ideology might capture ambit of concern better than economic ideology. Additional research can examine the specifically social component of ideology specifically. We also do not examine the relationship between ideology and ambit of concern for libertarians, a group that exhibits low general empathy, low universalism, but also low endorsement of the binding moral foundations that typify conservative ideology^34^. Additional research examining libertarians can better capture moral concern beyond the simple liberal–conservative dimension.

Finally, future research can address three issues pertaining to generalizability. First, future work can address manifestations of ideology at different time periods, as we cannot make overly broad claims about ideology based on data from this one (particularly polarized) point in history.^35^ Nonetheless, we do believe that the left–right distinction is quite robust throughout history even as it takes different forms from generation to generation. As Hibbing^31^ notes, John Stuart Mill called it “commonplace” for political systems to have “‘a party of order or stability and a party of progress or reform’… The antagonism between two primal mindsets certainly pervades human history: Sparta and Athens; optimates and populares; Roundheads and Cavaliers; Inquisition and Enlightenment; Protagonus and Plato; Pope Urban VIII and Galileo; Barry Goldwater and George McGovern; Sarah Palin and Hillary Rodham Clinton. The labels “liberal” or “leftist” and “conservative” or “rightist” may be relatively recent (etymologically they are typically assumed to date to the French Revolution, but they appear to be much older.”

The second issue pertaining to generalizability is sample representativeness. The present research used participants samples from platforms that were not necessarily representative and contained more political liberals than conservatives. To bolster our findings here that liberals relative to conservatives exhibit universalism relative to parochialism, we examined nationally representative data from the United States in the World Values Survey in a supplementary study (see Supplementary Methods). This study again demonstrated universalism–parochialism differences that manifested in conservatives (relative to liberals) exhibiting greater concern and preference for family relative to friends, the nation relative to the world, and humans relative to nonhumans. In addition, this data contain participants from multiple time periods from 1994 to 2014, suggesting—and speaking to the point raised in the previous paragraph—that the general patterns we find here are not specific to this singular moment in time.

The third issue concerns whether the findings here extend beyond the United States. The United States was the focus of the present work, but we acknowledge that other countries could show differing patterns. One possible reason is simply semantic—that is, “liberal” means different things in different countries, with the “Liberal Party” of Australia, for example, representing a center–right ideological position. In these studies, we address this issue by indicating for non-US participants in Studies 1a–2b’s instructions, that “liberal” refers to “progressives” and the political left whereas “conservatives” refers to “traditionalists” and the political right. Non-US participants in these studies, therefore, show the same pattern of results as US participants (see Supplementary Note 5). On the other hand, given differing historical trajectories and cultural norms around conceptualizations of friends, family, the nation, the world, humans, and nature these patterns may vary in certain contexts.

As one initial test of this, we examined countries from similar backgrounds as the United States including Canada, Australia, and Western Europe and also examined Eastern European countries, many of which have a history under a Communist regime. Whereas the countries similar to the United States showed the same pattern of results in terms of universalism versus parochialism, more variance emerged in the Eastern European countries (see Supplementary Table 1). Rather than attempt to explain all regional differences, we urge additional future research on cross-cultural comparisons. We continue to predict that, because conservative and liberal ideologies represent consistent sets of psychological differences^36^ in terms of motivational and cognitive processing styles, any ideology corresponding to a more closed and ordered mode of information processing will manifest in parochialism whereas any ideology corresponding to a more open and unstructured mode of information processing will manifest in universalism.

While people across the ideological spectrum all likely experience both centripetal and centrifugal forces in their ambits of concern^37^, liberals are more likely to distribute concern to outer circles and conservatives are more likely to distribute concern to inner circles. This suggests that it is the differential distribution of concern that contributes to and exacerbates moral debates across the political divide.

## Methods

All studies were institutional review board-approved by University of Southern California and Northwestern University and participants provided informed consent for each one.

### Study 1a, participants

Three thousand three hundred sixty-four participants (1791 male, M_age_ = 34.94, SD = 13.64) completed the study on the YourMorals.org website. At this website, participants first registered by completing basic demographic information including gender (0 = female, 1 = male), age (coded as blank if values > 95 to reduce fraudulent responses), and education (1 = some high school, 2 = currently in high school, 3 = completed high school, 4 = some college or university, 5 = currently in college, 6 = completed college or university, 7 = some graduate/professional school, 8 = currently in graduate or professional school, 9 = completed graduate or professional school; no answer = blank). In this study and all others conducted on YourMorals.org, removing participants who also completed one of the studies here or a study on YourMorals.org assessing a similar construct did not change the primary pattern of results.

Participants also indicated their political ideology (very liberal = 1, liberal = 2, slightly liberal = 3, moderate = 4, slightly conservative = 5, conservative = 6, very conservative = 7, and libertarian, do not know/not political, or other, the last three of which were excluded from analyses in this and all other studies). This ideology rating scale for YourMorals.org studies was also described to participants in a way that it could translate to countries with different conceptualizations of liberal/conservative:

“(The terms used in your country may differ. “Liberal” is intended to include the Left, progressives, and in some countries socialists. “Conservative” is intended to include the Right, traditionalists, and in some countries Christian Democrats.)”

There were 2619 liberals, 347 moderates, and 398 conservatives. In this study and all others conducted on YourMorals.org, some participants also separately indicated their ideology “on social issues” and “on economic issues” using the same options for the general political ideology question. Where these data are available from our participants, we also conduct primary analyses using measures of social and economic ideology and report them in Supplementary Note 3. Also see Supplementary Note 1 for more information on the samples obtained on YourMorals.org.

### Procedure

Participants completed the love of humanity scale^38^ that includes four subscales (1 = completely disagree, 7 = completely agree): romantic love (e.g., “My romantic partner and I are drawn to each other”), love for friends (e.g., “My friends and I look out for each other”), love for family (e.g., “My siblings and I love each other “warts and all”—we don’t censor ourselves around each other”), and love for all others (there are times in my life when I’ve felt strong feelings of love for all people, not just the specific people I’m close to”). Although all participants received all items, not all participants produced scores for every subscale (failing to answer any items pertaining to a specific subscale). Participants were included if they produced scores for at least one of these subscales, but not the others, resulting sometimes in slightly different degrees of freedom across subscales. This same specification applies to Studies 1b and 1c, which also contain subscales.

### Study 1b, participants

Thirteen thousand one hundred fifty-six participants (7113 male, M_age_ = 36.72 SD = 14.47) completed the study on the YourMorals.org website. During registration participants completed the same ideology, age, gender, and education measures described in Study 1a and in this sample consisted of 9625 liberals, 1684 moderates, and 1847 conservatives.

### Procedure

Participants completed the Schwartz Values Inventory^39^, which assesses various values that act as guiding principles for one’s life. Of importance were two measures in particular, one assessing values oriented toward the world as a whole—a set of items examining universalism, the concept of peace and equality for all (e.g., “A WORLD AT PEACE—free of war and conflict”)—and one item assessing nationalism (“NATIONAL SECURITY (protection of my nation from enemies”). Participants answered how important 58 value items were for their lives, and used a scale ranging from −1 (opposed to the value) to 0 (not at all important) to 7 (of supreme importance).

### Study 1c, participants

Fourteen thousand one hundred seventy-eight participants (8295 male, M_age_ = 36.70 SD = 14.05) completed the study on the YourMorals.org website. During registration participants completed the same ideology measure described in Study 1a and in this sample consisted of 10,674 liberals, 1534 moderates, and 1970 conservatives.

### Procedure

Participants completed the Identification With All Humanity Scale^40^ that asks how much people identify with their community, their country, and the world as a whole (e.g., “How much do you identify with (that is, feel a part of, feel love toward, have concern for) each of the following?” People in my community, people in my country, all humans everywhere). Participants used a 5-point scale (1 = not at all, 5 = very much) to answer nine questions for each of these entities—community, country, and all humans—that assessed identification with each one. We predicted that conservatism would predict identification with country, while liberalism would predict identification with all humanity. We had no hypothesis about identification with community.

### Study 2a, participants

Four thousand four hundred and twenty-eight participants (2269 male, M_age_ = 36.56 SD = 14.39) completed the study on the YourMorals.org website. Removing participants from analyses for which they were not required (see below) did not change the primary pattern of the results. During registration participants completed same ideology, age, gender, and education measures described in Study 1a and this sample consisted of 3136 liberals, 592 moderates, and 700 conservatives.

### Procedure

Participants began the task by reading the following instructions:

This is a study about pattern perception. On each of the next 30 screens, you are going to see two boxes. (Do not worry, each screen takes just a few seconds). In each box you will see some dots moving around. After 3 s, three buttons will appear. Please click on the appropriate button to indicate which box you like better. The buttons will disappear 5 s later, so you will have to make your judgments within that 5 s period.

That may seem like an odd judgment to make, but if you just relax and let yourself look back and forth between the two boxes, you will find yourself having some slight feelings in response. You will find yourself liking one box or the other a bit more. Do not think too much about the task, just go with your feelings.

Next, they proceeded to evaluate 30 screens, in which two animations were presented side by side (to view task access: http://yourmorals.org/dotspref_task.php; see Supplementary Fig. 1 for depiction of task). Each animation consisted of six dots that varied on two dimensions: (1) color diversity—all six dots were of the same color (nondiverse) or of all different colors (diverse); (2) looseness-tightness—the six dots either remained fixed as a single shape, a triangle (tight), moved individually, but retained the general shape of a triangle (mobile), or orbited around each other freely (loose). Animations were divided evenly into diverse and nondiverse color patterns as well as tight or loose movement patterns, and were randomly presented next to each other. After three seconds of the animation, participants were asked to select one of three options: “I prefer this one” (presented twice–once under each animation) or an option that said, “I have no preference at all.” If participants did not select an option within 5 s, text appeared informing participants they were too slow and asking them to try to click a button within 5 s the next time. After the presentation of these 30 screens, participants answered a question about how hard the task was and an open-ended question on what they thought the purpose of the task was.

For each participant, preference for color diversity was computed by subtracting the percentage preference for the nondiverse pattern from the percentage preference for the diverse pattern. Preference for looseness was computed by subtracting the percentage preference for the tight pattern from the combined percentage preference for the loose and mobile patterns.

Animations such as these are often spontaneously anthropomorphized^41,42^, and thus can be used as proxies for people’s perceptions of social ensembles. Furthermore, this method is in line with recent work that has used basic schematics and shapes to map low-level perceptual tendencies to meaningful political ideological differences^43,44^.

Originally, this study was designed for a different purpose, to study the relationship between political ideology and people’s feelings about superorganisms (emergent social entities), with the prediction that liberals would prefer more disordered ensembles, whereas conservatives would prefer more ordered ensembles. This study also compared displays of heterogeneously colored versus homogeneously colored ensembles, with the prediction that liberals would prefer more diversely colored ensembles and conservatives would prefer uniformly colored ensembles. Upon reexamining the study, we realized that it was relevant for our purpose to test our hypotheses about ideological differences in preference for moral circles of different types.

### Study 2b, participants

Two thousand and seventy-four participants (997 male, M_age_ = 34.87SD = 13.96) completed the study on the YourMorals.org website. Removing participants from analyses for which they were not required (see below) did not change the primary pattern of results. During registration participants completed the same ideology, age, gender, and education measures described in Study 1a and in this sample consisted of 1468 liberals, 270 moderates, and 336 conservatives.

### Procedure

The task participants perform was largely the same as in Study 2a with a few notable exceptions (to view task access: http://yourmorals.org/dotspref2_task.php; see Supplementary Fig. 2 for depiction of task): (1) Participants viewed 12 screens rather than 30. (2) Diversity in color was not manipulated (all the dots were the same color), but shape of the structure was 10 dots (rather than six in Study 2a) comprised the general shape of a triangle or circle (3). Looseness-tightness was manipulated on two, rather than three levels—the dots either remained fixed as a single shape, a triangle or circle (tight), or moved individually, but retained the general shape of a triangle or circle (loose). Animations were divided evenly into circle and triangle shapes as well as tight or loose movement patterns, and were randomly presented next to each other.

For each participant, preference for shape was computed by subtracting the percentage preference for the triangle shape from the percentage preference for the circle shape. Preference for looseness was computed by subtracting the percentage preference for the tight pattern from the percentage preference for the loose pattern.

Like Study 2a, this study was initially designed to test the prediction that liberals would prefer more disordered ensembles, whereas conservatives would prefer more ordered ensembles. The prediction regarding shape was that liberals relative to conservatives would prefer circles over triangles, manifesting a preference for egalitarianism over hierarchy. As with Study 2a, upon reexamining this study, we realized that it was relevant for our present hypotheses.

### Study 3a, participants

One hundred thirty-one United States residents (53 male, M_age_ = 35.82, SD = 13.76) were recruited from the Amazon Mechanical Turk (MTurk) marketplace for a small monetary reward. Although this sample size was simply chosen on the basis of similar past studies, a post hoc power analysis indicated that we had sufficient power (> 0.79) to detect the smallest correlation (in absolute value) found in our analyses below (*r* = −0.24). Participants completed the study using Qualtrics software, including an ideology measure that contained seven options (very liberal, liberal, slightly liberal, moderate, slightly conservative, conservative, very conservative). Participants were also asked about demographics including age and gender (coded as in Study 1a) and education, which participants were asked to enter in terms of years, with high school completion signifying 12. For participants who entered a nonnumerical response, we translated their response to a number using our best judgment (e.g., “some college” was translated to 14 years). Our sample included 64 liberals, 31 moderates, and 36 conservatives, and participants were only included in analyses if they completed the study in full. Sample size was determined based on attempts to maximize statistical power and was confined to participants who completed the study while it was available on the MTurk marketplace.

### Procedure

All participants completed a moral allocation task, in which participants allocated 100 “moral units” among the following 16 categories, pictured as increasingly large concentric circles (see full depiction of task in Supplementary Note 4): all of your immediate family; all of your extended family; all of your closest friends; all friends including more distant friends; all acquaintances; all people you have ever met; all people in your country; all people on your continent; all people on all continents; all mammals on all continents; all amphibians, reptiles, mammals, fish, and birds; all animals on earth including paramecia and amoebae; all animals in the universe, including alien lifeforms; all living things in the universe including plants and trees; all natural things in the universe including inert entities such as rocks; all things in existence. Participants read the following instructions:

In this section, we would like to think about your capacity to help, to give, to be charitable, to show empathy, and to be generous—in other words, your capacity to behave morally. We can think about people having different amounts of moral units—like currency—that they can spend on others and can allocate to different moral circles. Some people devote all of their moral units to one circle whereas others try to divide up their moral units amongst multiple circles. Again, by moral circle, we mean the circle of people or other entities in which you are concerned about right and wrong done toward them.

We also explained to participants that these categories were non-overlapping such that giving to one category (e.g., extended family) would not include an inclusive category (e.g., immediate family). Participants completed two iterations of this task (order randomized). In one, they were asked to allocate moral units how one should ideally divide them. In the other, they were asked to divide them as they personally do so in their daily lives. These allowed us to assess differences between actual and ideal moral allocation, but no meaningful differences emerged. The categories allowed us to create composite moral allocation scores for humans only (average of units allocated to the first nine categories) and for nonhumans (average of units allocated to the last seven categories). In addition, participants also completed a more qualitative measure of the extent of their moral circle by clicking on rungs extending outward and representing the same categories as in the moral allocation task (see Supplementary Note 4). This measure allowed us to create heatmaps to visualize the relative sizes of liberals’ and conservatives’ moral circles. This task was also counterbalanced in presentation with the moral allocation task, and no order effects emerged.

### Study 3b, participants

Two hundred sixty-three United States residents (173 male, M_age_ = 28.02, SD = 9.13) were recruited from the Amazon Mechanical Turk (MTurk) marketplace for a small monetary reward and completed the study using Qualtrics software, including the same ideology, age, gender, and education measures used in Study 3a. Our sample included 176 liberals, 45 moderates, and 42 conservatives, and participants were only included in analyses if they completed the study in full. Sample size was determined by attempting to double the sample size of Study 3a, to ensure sufficient power.

### Procedure

Participants completed the same personal moral allocation task as in Study 3a, with one alteration. Participants were told that they could allocate any amount to any group, and any amount overall. Participants varied greatly in their total allocation of units to all categories, from 10 to 10^53^. Although we made an a priori decision not to exclude outliers, all results described below remain the same when excluding the one participant whose allocations fall outside of 3SD of the mean. (We included this participant in analyses because our primary analyses involve proportions, which are constrained between 0 and 1 for all participants.).

To analyze moral allocation to humans versus nonhumans, we computed a proportion score for humans and nonhumans separately. To compute the human allocation proportion score, we summed for each participant the nine categories pertaining to humans exclusively and divided by the total units allocated to all categories. To compute the nonhuman allocation proportion score, we summed for each participant the seven categories pertaining to nonhumans and divided by the total units allocated to all categories.

### Reporting summary

Further information on research design is available in the Nature Research Reporting Summary linked to this article.

## Supplementary information

Supplementary Information

Reporting Summary

Source Data

## Data Availability

The datasets generated during and analyzed during this study are available from the corresponding author on reasonable request. A reporting summary for this Article is available as a Supplementary Information file.
