# Supplementary material for: Ideological differences in the expanse of the moral circle
Source: Nat Commun. 2019 Sep 26;10:4389. doi: 10.1038/s41467-019-12227-0 (PMC6763434; doi:10.1038/s41467-019-12227-0)
Supplement: Supplementary file 1 — Supplementary Information [file 41467_2019_12227_MOESM1_ESM.pdf]

Supplementary Materials

Ideological Differences in the Expanse of the Moral Circle

Waytz et al.

Supplementary Note 1. Additional Information on YourMorals.org samples

For Study 1a and all other studies on YourMorals.org, sample size was determined by including everyone who completed the primary study instruments as of January 14, 2011. In this study and all others conducted on YourMorals.org, we did not analyze data from people who completed the study more than once.

The data files produced for Studies 1a and 1c excluded participants who failed to complete 20% of the items on the critical measure from which subscales were derived. This exclusion rule was implemented by the second author prior to analyses conducted by the first author, and was then kept in place for these studies so as not to alter the exclusion rule after looking at the data.

Supplementary Note 2. Exploration of Quadratic Effects

Study 1a

The means for love of friends appeared curvilinear, so we tested for a quadratic effect by regressing love of friends on the political ideology variable at Step 1 and on the square of the political ideology variable at Step 2. Step 1 revealed a significant linear effect, *ß* =-.065, $t(3360)=3.76$, $p<.001$. Step 2 revealed a significant linear effect, *ß* =-.22, $t(3359)=3.09$, $p=.002$, and a significant quadratic effect, *ß* =.16, $t(3359)=2.23$, $p=.026$. However, a quadratic effect does not necessarily indicate a U-shaped relationship[1], so we investigated this data further. Further inspection of the data examined whether the data are better characterized in terms of a linear relationship (suggesting that as conservatism increases parochialism increases) or U-shaped relationship (suggesting that as conservatism increases parochialism increases to a point, then reverses). We examined these relationships comparing the standardized beta of linear effect to that of the quadratic effect, and per the suggestion of Simonsohn and Nelson[2], conducting

separate linear regressions—one up to the point where the value of the outcome variable maxes out (in this case, where it bottoms out, for the value of 5=slightly conservative), and another from that point onwards.

The first analysis involved multiplying the political ideology variable by -1 to produce a variable that would produce the same mathematical sign (positive) as the variable indicative of the quadratic effect. We then standardized both the new political ideology variable and the existing political ideology squared variable (within the baseline condition only), and compared their effects on love of friends, using a custom hypothesis test in SPSS software. This test allowed us to contrast the linear effect (coded as -1) to the quadratic effect (coded as 1), which revealed a significant (p<.001) difference, suggesting that the linear effect was significantly greater than the quadratic effect

The second analysis involved regressing love of friends on three variables per Simonsohn and Nelson[2]: a first variable representing political ideology up until the point that love of friends bottoms out (very liberal recoded as -4, liberal recoded as -3, slightly liberal recoded as -2, moderate recoded as -1, slightly conservative recoded as 0, conservative recoded as 0, and very conservative recoded as 0), a second variable representing political ideology from this point onwards (very liberal recoded as 0, liberal recoded as 0, slightly liberal recoded as 0, moderate recoded as 0, slightly conservative recoded as 0, conservative recoded as 1, and very conservative recoded as 2), and a dummy variable (very liberal recoded as 0, liberal recoded as 0, slightly liberal recoded as 0, moderate recoded as 0, slightly conservative recoded as 0, conservative recoded as 1, and very conservative recoded as 1). This regression revealed a significant effect for political ideology values up until the bottom-out point (slightly conservative), $ß$ =-.08, t(3358)=3.98, p<.001 and a non-significant effect for political values

from this point onwards, *ß* =.03, $t(3358)=0.59$, $p=.56$. Thus, we do not conclude a significant curvilinear relationship. Based on these two analyses, we suggest the data are better characterized in terms of a linear relationship between political ideology and love of friends.

Supplementary Note 3. Primary Analyses Using Social and Economic Ideology

Study 1a

Considerably fewer participants completed measures of social and economic ideology than in the primary analyses, so these results should be interpreted with caution. As with the primary analysis, non-significant relationships emerged between romantic love and social ideology and economic ideology, $r(306)=-.09$, $p=.12$ and $r(298)=-.06$, $p=.34$, respectively. Non-significant relationships also emerged for the relationship between love of family and social ideology and economic ideology, with social ideology producing a pattern opposite of the pattern with general ideology, $r(315)=-.024$, $p=.67$ and $r(307)=.01$, $p=.93$. Social and economic ideology both produced a relationship between liberal ideology and love of friends yet this relationship was only significant for social ideology, $r(314)=-.11$, $p=.05$ and $r(306)=-.04$, $p=.52$, respectively. Both social and economic ideology produced significant relationships between liberal ideology and love of all others $r(315)=-.33$, $p<.001$ and $r(307)=-.32$, $p<.001$, respectively.

Study 1b

As with Study 1a, considerably fewer participants completed measures of social and economic ideology than in the primary analyses and should be interpreted with caution. Nonetheless, all primary findings replicated. Social and economic ideology both produced a significant relationship between liberal ideology and universalism, $r(1283)=-.40$, $p<.001$ and $r(1245)=-.41$, $p<.001$, respectively. Social and economic ideology both produced a significant

relationship between conservative ideology and nationalism, $r(1271)=.43$, $p<.001$ and $r(1235)=.39$, $p<.001$, respectively.

Study 1c

As with Studies 1a-1b, considerably fewer participants completed measures of social and economic ideology than in the primary analyses and should be interpreted with caution. Interestingly, in this limited sample, there seems to be a divergence between social ideology and economic ideology such that social ideology has virtually no relationship to identification with community, $r(639)=.003$, $p=.93$, whereas—contrary to findings for general ideology—economic ideology shows an association between liberal ideology and identification with community, $r(616)=-.09$, $p=.028$. As with general ideology, social and economic ideology both produced a relationship between conservative ideology and identification with country, $r(639)=.15$, $p<.001$ and $r(616)=.07$, $p=.081$ (marginal), respectively. Also, as with general ideology, social and economic ideology both produced a relationship between liberal ideology and identification with all humanity, $r(639)=-.39$, $p<.001$ and $r(616)=-.40$, $p<.001$, respectively.

Study 2a

Social and economic ideology both produced a relationship between liberal ideology and preference for looseness relative to tightness, $r(1907)=-.20$, $p<.001$ and $r(1845)=-.18$, $p<.001$, respectively. Social ideology did not produce a significant relationship for preference for diversity of color, $r(1907)=-.03$, $p=.22$, yet economic ideology produced a significant relationship between liberalism and preference for diversity, $r(1845)=-.06$, $p=.01$.

Study 2b

As with Studies 1a-1c, considerably fewer participants completed measures of social and economic ideology than in the primary analyses and should be interpreted with caution. Social

and economic ideology both produced a relationship between liberal ideology and preference for looseness relative to tightness, $r(796)=-.23$, $p<.001$ and $r(777)=-.18$, $p<.001$, respectively. Neither social ideology nor economic ideology produced significant relationship for shape preference, $r(796)=-.02$, $p=.61$ and $r(777)=-.05$, $p=.19$, respectively.

Supplementary Figure 1.

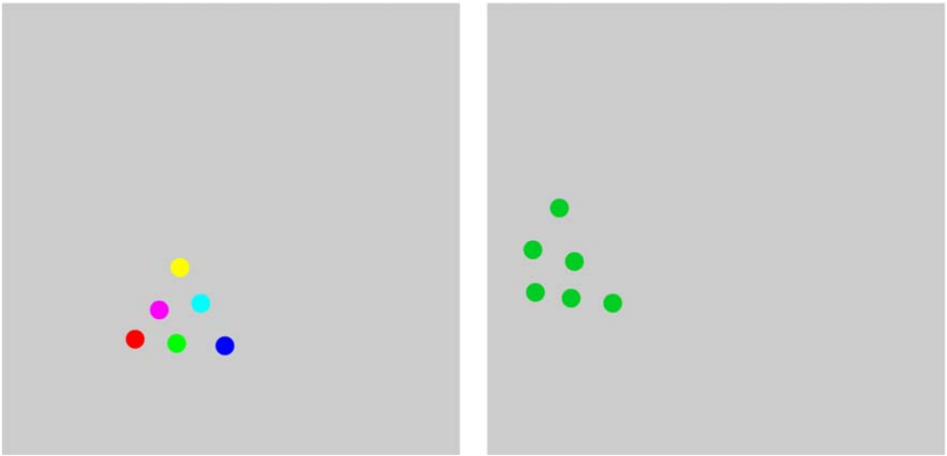

Depiction of task, Study 2a.

Supplementary Figure 2.

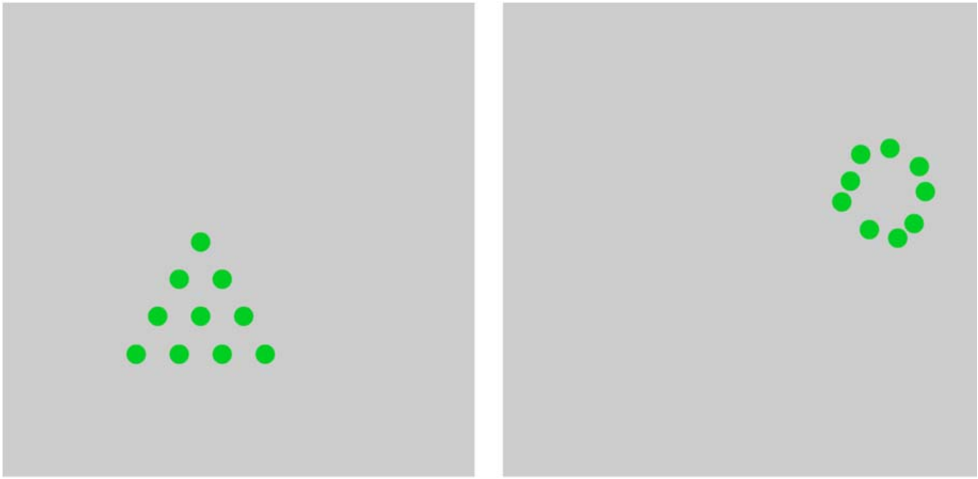

Depiction of task, Study 2b.

Supplementary Note 4.

Instructions for Circle Tasks Used in Studies 3a-3b

On this page, we would like you to indicate the extent of your moral circle. By moral circle, we mean the circle of people or other entities for which you are concerned about right and wrong done toward them. This depiction demonstrates that people have different types of moral circles. At the innermost circle, some people care about their immediately family only, and at the outermost circle, people care about the entire universe--all things in existence. Please use the following scale and select a location that depicts the extent of your moral circle.

1 - all of your immediate family
2 - all of your extended family
3 - all of your closest friends
4 - all of your friends (including distant ones)
5 - all of your acquaintances
6 - all people you have ever met
7 - all people in your country
8 - all people on your continent
9 - all people on all continents
10 - all mammals
11 - all amphibians, reptiles, mammals, fish, and birds
12 - all animals on earth including paramecia and amoebae
13 - all animals in the universe, including alien lifeforms
14 - all living things in the universe including plants and trees
15 - all natural things in the universe including inert entities such as rocks
16 - all things in existence

Please click on a number that depicts the extent of your moral circle. Note that in this scale, the number you select includes the numbers below it as well. So, if you select 10 (all mammals), you are also including numbers 1-9 (up to 'all people on all continents') in your moral circle.

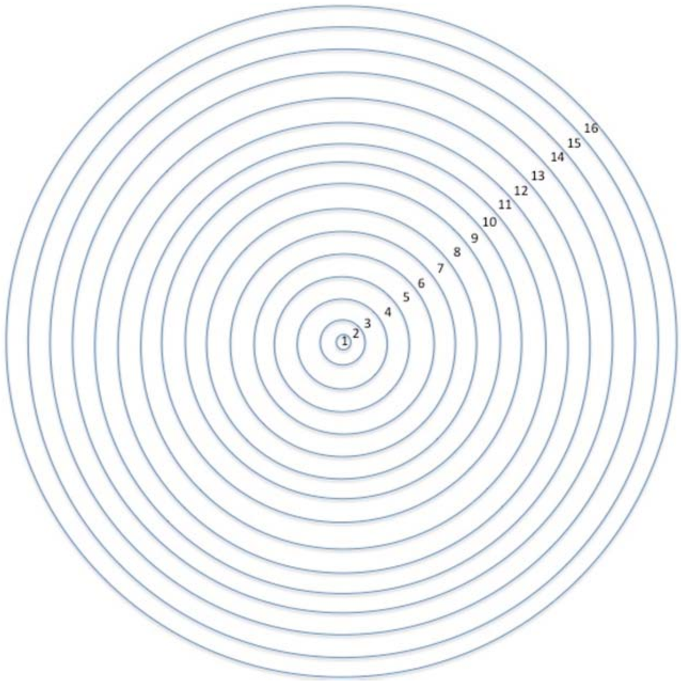

Supplementary Note 5.

Correlations between ideology and constructs of interest by US vs. non-US nationality (Studies 1a-2b)

Study 1a (27% non-USA participants)

Love of family (non-USA): $r(896)=.019$, $p=.58$
Love of family (USA): $r(2464)=.077$, $p<.001$
Love of friends (non-USA): $r(896)=-.069$, $p=.04$
Love of friends (USA): $r(2462)=-.07$, $p<.001$
Love for all others (non-USA): $r(896)=-.15$, $p<.001$
Love for all others (USA): $r(2464)=-.22$, $p<.001$

Study 1b (32% non-USA participants)

Nationalism (non-USA): $r(4235)=-.33$, $p<.001$
Nationalism (USA): $r(8917)=-.45$, $p<.001$
Universalism (non-USA): $r(4199)=.42$, $p<.001$
Universalism (USA): $r(8829)=.48$, $p<.001$

Study 1c (22% non-USA participants)

Identification with country (non-USA): $r(3122)=.15$, $p<.001$
Identification with country (USA): $r(11052)=.31$, $p<.001$
Identification with humanity (non-USA): $r(3122)=-.29$, $p<.001$
Identification with humanity (USA): $r(11052)=-.36$, $p<.001$

Study 2a (23% non-USA participants)

Preference for looseness versus tightness (non-USA): $r(1006)=-.20$, $p<.001$
Preference for looseness versus tightness (USA): $r(3418)=-.20$, $p<.001$

Study 2b (25% non-USA participants)

Preference for looseness versus tightness (non-USA): $r(518)=-.21$, $p<.001$
Preference for looseness versus tightness (USA): $r(1552)=-.13$, $p<.001$

Supplementary Methods.

World Values Study

This study employs the World Values Survey (WVS)[3]. The WVS is a broad international questionnaire administered from 1981 to the present that surveys representative samples from different countries (including the United States) about their values, beliefs, and opinions. We used this data to assess whether the general patterns of results we found in the primary studies replicated in a representative sample.

Method

Participants. To conceptually replicate the findings from the primary studies, we used data only from United States respondents (unweighted), which included 6,223 individuals (2,983 male; M_{age}=47.32, *SD*=17.22). We used all responses to the pertinent questions, which were administered during waves of the survey spanning from 1994 to 2014, described below.

Ideology was assessed on a 10-point scale asking, “In political matters, people talk of ‘the left’ and ‘the right.’ How would you place your views on this scale, generally speaking?” (1=Left, 10=Right) and responses were excluded if participants responded with any other option. The following frequencies emerged for each response: 1=135, 2=143, 3=375, 4=389, 5=1956, 6=1085, 7=606, 8=589, 9=303, 10=279.

Procedure. Our goal was to assess the basic patterns that emerged between liberals and conservatives on moral concern toward friends relative to family, the world relative to the nation, and humans relative to nonhumans. To do this, we identified items in the WVS that captured each construct. Given that we were using an existing dataset, the items were not always perfect proxies for these constructs, but we used the best items available to assess the patterns of data we found in our experiments.

For friends versus family, we used two items that followed this prompt, “For each of the following aspects, indicate how important it is in your life.” One item completed this prompt by asking about, “friends” and the other item asked about, “family.” Response options were: 1=Very important, 2=Rather important, 3=Not very important, 4=Not at all important (for these items and all other items, any other responses such as “don’t know” or “not applicable” were deleted before analysis). We computed a friends-versus-family score by subtracting the family item from the friends item.

For world versus nation, we used two items that followed this prompt, “People have different views about themselves and how they relate to the world. Using this card, would you tell me how strongly you agree or disagree with each of the following statements about how you see yourself?” One item completed this prompt with, “I see myself as a world citizen” and the other completed this prompt with “I see myself as citizen of the [country] nation.” Response options were: 1=Strongly Agree, 2=Agree, 3=Disagree, 4=Strongly Agree. We computed a world-versus-nation score by subtracting the world item from the nation item.

For nonhumans versus humans, we used two items that followed this prompt, “Now I will briefly describe some people. Using this card, would you please indicate for each description whether that person is very much like you, like you, somewhat like you, not like you, or not at all like you?” One item completed the prompt with, “Looking after the environment is important to this person; to care for nature” and the other completed the prompt with “It is important to this person to help the people nearby; to care for their well-being.” Response options were: 1=very much like me, 2=like me, 3=somewhat like me, 4=a little like me, 5=not like me, 6=not at all like me. We computed a nonhumans-versus-humans score by subtracting the nonhumans item from the humans item.

Results

Correlations revealed that political ideology correlated marginally significantly with the friends-versus-family score, $r(5835)=.024$, $p=.065$ and significantly with the world-versus-nation ($r(3276)=.23$, $p<.001$) score and nonhumans-versus-humans score ($r(1187)=.14$, $p<.001$). The patterns of these results reveal that conservatism is associated with valuing family relative to friends, the nation relative to the world, and humans relative to nonhumans. In multiple regressions including education, age, and sex, the effect of politics remained a stable predictor of all three variables ($p=.056$ for friends-versus-family, $p<.001$ for world-versus-nation and nonhumans-versus-humans). These findings thus support the results established in the primary studies in the manuscript, in a more representative American sample.

Exploratory Analysis

As an exploratory analysis, we conducted the same analyses as above in regions with similar and dissimilar cultural pasts to the United States. Specifically, we conducted these analyses in regions that, like the United States are relatively WEIRD (Western Educated Industrialized Rich Democratic)[4] and a set of countries in Eastern Europe that are relatively less WEIRD and that have historically existed under a Communist regime. We focused on five regions, based on existing research conducted with World Values Survey data, by Diez-Nicolas[5], who writes:

> “Countries were grouped in the following cultural-territorial regions: Anglo-Saxon: Australia, Canada, Great Britain, Ireland, New Zealand, North Ireland, USA; West European Catholic: Andorra, Belgium, France, Italy, Luxemburg, Malta, Netherlands, Portugal, Spain, Switzerland; West European Protestant: Austria, Denmark, Estonia, Finland, Germany, Iceland, Latvia, Lithuania, Norway, Slovakia, Sweden; East European Christian: Czech Rep., Hungary, Poland, Slovenia; European Orthodox: Armenia, Belarus, Bulgaria, Croatia, Cyprus, Georgia, Greece, Macedonia, Moldova, Romania, Russia, Serbia, Serbia and Montenegro, Ukraine.”

Countries that did not have data for this analysis were Ireland, Northern Ireland, Belgium, Luxemburg, Malta, Portugal, Austria, Denmark, Iceland, Greece, and Macedonia (in addition,

the country codes for Serbia and Montenegro were separately used for Serbia and Montenegro, and the country code for United Kingdom was used for Great Britain).

We did not include the United States in our analysis of the Anglo-Saxon region, but predicted this region would resemble most closely the United States. We also predicted that the West European Catholic and West European Protestant regions would resemble the United States whereas the Eastern European regions (East European Christian and European Orthodox) would differ because of differing historical and political trajectories. We focused on these broad sets of regions, which include Western and Eastern Europe because of countries' geographic proximity to one another.

As Supplementary Table 1 shows, as predicted, the patterns for each variable are identical to the United States and statistically significant for the Anglo-Saxon, West European Catholic, and West European Protestant regions. For the other two regions, the only pattern that significantly replicates that of the United States is the association between political ideology and the world-versus-nation score for the East European Christian region, with the correlation reversing for all variables for the East European Orthodox region. As stated in the main text, given the many possible explanations for these patterns, we urge future research on the topic.

Supplementary Table 1. **Correlations by region.**

**Region**	**friends-versus-family**	**world-versus-nation**	**nonhumans-versus-humans**
**Anglo-Saxon**	.040	.123	.034
	<.001	<.001	0.038
	10673	4278	3816
**West European Catholic**	.048	.172	.053
	<.001	<.001	<.001
	13122	6183	7547

**West European Protestant**	.032	.089	.053
	<.001	<.001	<.001
	17503	8589	5558
**East European Christian**	-0.016	.049	0.011
	0.145	0.004	0.608
	8397	3559	2223
**East European Orthodox**	-.021	-0.01	-.048
	<.001	0.263	<.001
	31027	13212	6418

Note: These variables listed here are the friends-versus-family score, the world-versus nation score, and the nonhumans-versus humans score. In each cell is the correlation between ideology and the variable at the top of the column, below it is the p-value, and below the p-value is the sample size. Positive correlations indicate the same relationship as shown in the United States.

Supplementary References

1. Lind, J. T., & Mehlum, H. With or without U? the Appropriate Test for a U-Shaped Relationship. *Oxf. Bull. Econ. Stat.* ***72***, 109-118 (2010).
2. Simonsohn, U. & Nelson, L. *Data from "Thirty-somethings are shrinking and other u-shaped challenges*." http://datacolada.org/2014/09/17/27-thirty-somethings-are-shrinking-and-other-u-shaped-challenges/ (2014).
3. World Values Survey Association. European and World Values Surveys four-wave integrated data file, 1981–2004, v. 20060423 [data set]. Stockholm, Sweden: http://www.worldvaluessurvey.org (n.d.).
4. Henrich, J., Heine, S. J. & Norenzayan, A. The weirdest people in the world?. *Behav. Brain. Sci.* ***33***, 61-83 (2010).
5. Diez-Nicolas, J. Cultural differences on values about conflict, war, and peace. *World Values Research*, ***3***, 1-20 (2010).
